# Adsorption of β-glucosidases in two commercial preparations onto pretreated biomass and lignin

**DOI:** 10.1186/1754-6834-6-165

**Published:** 2013-11-25

**Authors:** Mai Østergaard Haven, Henning Jørgensen

**Affiliations:** 1 DONG Energy A/S, Kraftværksvej 53, Fredericia 7000, Denmark; 2 Department of Geosciences and Natural Resource Management, University of Copenhagen, Faculty of Science, Rolighedsvej 23, Frederiksberg C 1958, Denmark

**Keywords:** Adsorption, Beta-glucosidase, BSA, Cellulase preparations, Lignocellulosic bioethanol, PEG, pNPG

## Abstract

**Background:**

Enzyme recycling is a method to reduce the production costs for advanced bioethanol by lowering the overall use of enzymes. Commercial cellulase preparations consist of many different enzymes that are important for efficient and complete cellulose (and hemicellulose) hydrolysis. This abundance of different activities complicates enzyme recycling since the individual enzymes behave differently in the process. Previously, the general perception was that β-glucosidases could easily be recycled via the liquid phase, as they have mostly been observed not to adsorb to pretreated biomass or only adsorb to a minor extent.

**Results:**

The results from this study with Cellic® CTec2 revealed that the vast majority of the β-glucosidase activity was lost from the liquid phase and was adsorbed to the residual biomass during hydrolysis and fermentation. Adsorption studies with β-glucosidases in two commercial preparations (Novozym 188 and Cellic® CTec2) to substrates mimicking the components in pretreated wheat straw revealed that the *Aspergillus niger* β-glucosidase in Novozym 188 did not adsorb significantly to any of the components in pretreated wheat straw, whereas the β-glucosidase in Cellic® CTec2 adsorbed strongly to lignin.

The extent of adsorption of β-glucosidase from Cellic® CTec2 was affected by both type of biomass and pretreatment method. With approximately 65% of the β-glucosidases from Cellic® CTec2 adsorbed onto lignin from pretreated wheat straw, the activity of the β-glucosidases in the slurry decreased by only 15%. This demonstrated that some enzyme remained active despite being bound. It was possible to reduce the adsorption of Cellic® CTec2 β-glucosidase to lignin from pretreated wheat straw by addition of bovine serum albumin or poly(ethylene glycol).

**Conclusions:**

Contrary to the β-glucosidases in Novozym 188, the β-glucosidases in Cellic® CTec2 adsorb significantly to lignin. The lignin adsorption observed for Cellic® CTec2 is usually not a problem during hydrolysis and fermentation since most of the catalytic activity is retained. However, adsorption of β-glucosidases to lignin may prove to be a problem when trying to recycle enzymes in the production of advanced bioethanol.

## Background

Processes for production of advanced bioethanol from lignocellulosic materials by biochemical conversion are now operated on a large scale. In the Inbicon demonstration plant (Kalundborg, Denmark), DONG Energy produces bioethanol, C5 molasses and lignin pellets, mainly from wheat straw, and the bioethanol produced here is sold at service stations throughout Denmark [1]. A number of other companies have similar projects ongoing - among these are Abengoa [2], Chemtex/Beta Renewables [3] and DuPont [4].

Despite recent advances in developing new and more efficient enzyme preparations [5,6], enzymes (cellulases and hemicellulases) still contribute significantly to the production costs. According to Humbird *et al.,* the enzyme cost in their model accounts for more than 15% of the minimum ethanol selling price [7]; older publications have reported the enzyme costs to be between 9% and 33% of the minimum ethanol selling price depending on process configuration [8,9]. One way to reduce this cost could be by recycling the enzymes. The most important factor that has impeded implementation of enzyme recycling is adsorption of enzymes onto residual substrate (primarily lignin). Tu *et al*. demonstrated that in experiments with organosolv-pretreated lodgepole pine with low lignin content the cellulases could be recycled four times with hydrolysis yields above 80%, whereas cellulases from hydrolysis of steam-exploded lodgepole pine could not be recycled in a similar manner (yield after first recycling was only approximately 60%) [10].

Over the years, several methods have been suggested for enzyme recycling, for example contact between the lignin-rich residue after hydrolysis/fermentation and new pretreated biomass or different methods for recycling liquid. In some cases, the recycling of liquid has been combined with pH adjustment or alkaline wash to desorb cellulases and/or ultrafiltration to concentrate the cellulases prior to recycling [11-18].

Lignocellulosic biomass contains three main components - cellulose, hemicellulose and lignin. During pretreatment, the structure of the three components is altered depending on the type of pretreatment. Hydrothermal pretreatment, dilute acid pretreatment and steam explosion degrade and solubilize some of the hemicellulose and rearrange or alter the lignin structure within the plant cell wall. Organosolv pretreatment removes a major part of the lignin and some hemicellulose [19]. Common characteristics for all pretreatment methods are that they rearrange and in some cases remove parts of the cell wall structure and render it more accessible for enzymatic degradation by cellulases. Lignin is a large random polymer that contains a wide range of functional groups (for example, hydroxyl, methoxyl and carbonyl) and chemical bonds between them (for example, β-aryl ether structure and β-β bonds). Lignin is generally composed of three major monolignols differing in the substitution pattern of methoxy groups on the aromatic ring: *p-*coumaryl alcohol (H units), coniferyl alcohol (G units) and sinapyl alcohol (S units). The composition of lignin - the ratio between H, G and S units - depends on the type of plant. Generally, softwood (like spruce) almost exclusively contains G units, whereas for instance grasses (like wheat straw) contain all three monolignols [20]. Because the pretreatment changes the chemical composition and structure of the pretreated biomass, this will also affect the adsorption of the enzymes during hydrolysis and fermentation and thereby probably also the recyclability of the enzymes [13,21].

When lignocellulosic biomass is enzymatically hydrolyzed using commercial cellulase preparations, many different activities are present to ensure efficient hydrolysis of cellulose (and hemicellulose). These include different cellulases (endo- and exoglucanases), β-glucosidases, xylanases [22,23], esterases [24], swollenins or expansins [25], and oxidative enzymes [26]. This diversity and the multiple enzyme activities complicate the recycling process because each of these individual enzymes have different physical properties, such as stability and adsorption/desorption behavior.

The phenomenon of adsorption of cellulases onto cellulose is well investigated. During hydrolysis of biomass, cellulases are generally adsorbed to the pretreated substrate rapidly [27-30]. Singh *et al*. reported that cellulases were adsorbed to Avicel and potato pulp within the first 10 min of contact at 30°C [28]. Adsorption of cellulases to cellulose is necessary to bring the enzymes in close contact with the insoluble substrate, thereby enabling the catalytic process to take place. In some cases, the cellulases are reported to desorb as the cellulose and hemicellulose are hydrolyzed, [31] whereas in other cases they are reported to stay adsorbed [32]. The latter is likely related to unproductive binding or adsorption of cellulases to lignin through hydrophobic interactions, which is also a well-known phenomenon in hydrolysis lignocellulosic materials. The unproductive binding of cellulases can be reduced or avoided by addition of protein [33], surfactants [34-36] or surfactant precursors [37-39].

Although involved in cellulose degradation, β-glucosidases are not true cellulases. Their substrate is cellobiose, which is soluble, unlike cellulases that degrade insoluble cellulose. Generally, β-glucosidases have been thought (and in some cases also demonstrated) to stay free in solution during hydrolysis and fermentation. For example, Berlin *et al*. described that the electrophoresis band corresponding to β-glucosidase activity in the liquid phase did not change during hydrolysis [31]. However, others have reported that β-glucosidases can also adsorb to pretreated biomass. Yang and Wyman [33] demonstrated that β-glucosidases from Novozym 188 adsorbed to dilute acid-pretreated corn stover and Pareek *et al*. [40] also observed that this β-glucosidase could adsorb to different lignin and hemicellulose preparations. Pribowo *et al*. [41] reported that β-glucosidase activity was found in both the liquid and solid phase, indicating that β-glucosidases can adsorb to the solid particles of pretreated biomass. Várnai *et al*. have demonstrated that β-glucosidases can adsorb to some substrates but to a smaller extent than observed for cellulases [42].

The adsorption and desorption as well as the stability of different enzymes in the cellulase mixture are properties of the individual enzymes. Therefore, the recovered enzyme mixture after hydrolysis and fermentation is likely to have a different activity profile compared to the original mixture and may even be devoid of essential activities [10,43].

**Figure 1:**
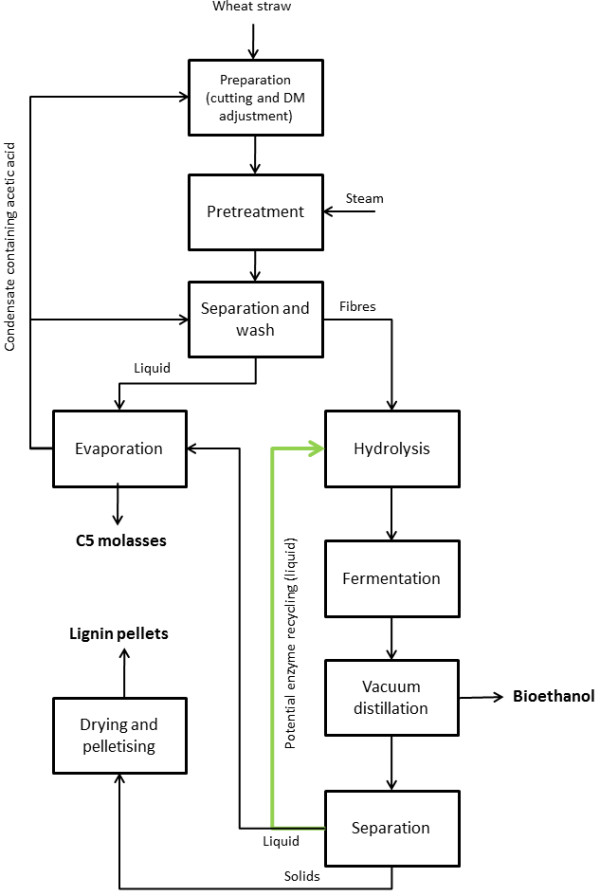
**Overview of the process streams in the Inbicon demonstration plant, including a stream for potential enzyme recycling after vacuum distillation.** Adapted with permission from [1].

The initial aim of this work was to recycle (part of) the enzymes through the liquid phase after hydrolysis, fermentation and vacuum distillation (Figure 1). However, our initial experiments with Cellic® CTec2 showed that β-glucosidase activity in the liquid phases was almost absent, and therefore β-glucosidases could not be recycled by this method. Here, we present data from experiments aiming at describing the adsorption of β-glucosidases from two commercially available enzyme preparations (Novozym 188 and Cellic® CTec2, both from Novozymes, Bagsværd, Denmark) to different components in pretreated wheat straw as well as other pretreated biomasses. Because the enzyme preparations were not pure β-glucosidase preparations, the β-glucosidase adsorption was measured indirectly by comparing the amount of free β-glucosidase activity in the liquid phase of samples incubated with and without substrate. The relative amount of free β-glucosidase activity was calculated from these two values.

## Results and discussion

### Hydrolysis and fermentation of pretreated wheat straw

Initial experiments with recycling of enzymes after hydrolysis and fermentation of pretreated wheat straw at 25% dry matter (DM) with Cellic® CTec2 showed that, surprisingly, only a small amount of β-glucosidase activity was free in solution (less than 1% of the initial activity). Interestingly, there was no sign of cellobiose build-up during the process as would have been the case if the β-glucosidases were inactivated. The activity measurements were supported by SDS-PAGE, which showed that the band around 110 to 120 kDa typically associated with β-glucosidases was indeed absent from the liquid (the *Aspergillus niger* β-glucosidase in, for example, Novozym 188 has a molecular weight of approximately 120 kDa). Therefore, it seemed that the β-glucosidases were adsorbed to the pretreated biomass but still catalytically active. This was not in line with the general perception that β-glucosidases either do not absorb onto pretreated biomass [10,13,31] or only adsorb to a minor extent [42]. Additional experiments where the solid residue after hydrolysis and fermentation was washed with buffer showed that it was not possible to desorb the β-glucosidases from the pellet (confirmed by both measurements of β-glucosidase activity and SDS-PAGE, data not shown). Furthermore, addition of cellobiose to suspensions of the residual solids in 0.1 M Na-acetate buffer (pH 4.8) demonstrated that the β-glucosidases were still active as the cellobiose was rapidly hydrolyzed to glucose. These initial results indicated that the β-glucosidases in Cellic® CTec2 adsorbed strongly to the residual solids but were still catalytically active. Previously, changes in pH have been demonstrated to desorb enzymes [44-46]. However, in this case, changes of pH in the range from 5 to 10 did not lead to a significant desorption of β-glucosidase activity. The highest amount of free β-glucosidase activity was observed when pH was adjusted to 9 or 10 for a maximum of 15 min, in which case less than 2% of initial activity was recovered.

### β-glucosidase adsorption experiments

The adsorption of β-glucosidases from Cellic® CTec2 to different substrates was compared to the adsorption of β-glucosidases in Novozym 188. Novozym 188 contains β-glucosidases from *A. niger* and has for many years been the classical reference β-glucosidase extensively used to supplement *Trichoderma reesei* cellulases (for example, Celluclast®) with β-glucosidase activity for hydrolysis of lignocellulosic biomass. Initial experiments demonstrated that after 1 h incubation with lignin from pretreated wheat straw at 33°C, the amount of free β-glucosidase activity from Cellic® CTec2 had stabilized (measurement of free β-glucosidase activity in the supernatant every 10 min for a total of 100 min). The two enzyme preparations were incubated (1 h, 33°C) with different substrates as described for the individual experiments and the β-glucosidase activity in the supernatant was measured and compared with the activity of a reference without added substrate.

### β-glucosidase adsorption to pretreated wheat straw

**Figure 2:**
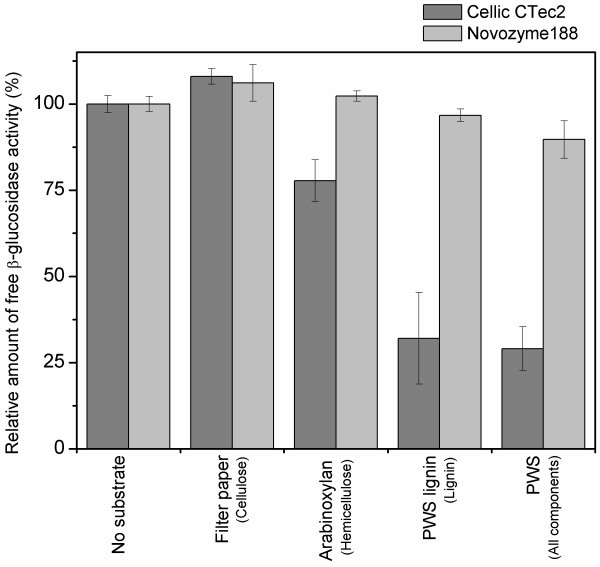
**Amount of free β-glucosidase activity in experiments with addition of different substrates.** No substrate (reference); filter paper (cellulose); arabinoxylan (hemicellulose); lignin, PWS lignin (lignin from pretreated wheat straw) and PWS (pretreated wheat straw containing a mixture of cellulose, hemicellulose and lignin). Substrate loadings: cellulose, 24 mg/mL; hemicellulose, 2 mg/mL; lignin, 15 mg/mL; and pretreated wheat straw, 48 mg/mL. Enzyme dosage: 500 nkat/mL. The activities are relative to the control without added substrate for each enzyme.

A comparison was made of the adsorption of β-glucosidases from Cellic® CTec2 and Novozym 188 to different substrates mimicking the individual components in pretreated biomass. The substrates were cellulose (filter paper), hemicellulose (arabinoxylan) and enzymatically purified lignin from pretreated wheat straw as well as pretreated wheat straw containing a mixture of cellulose, hemicellulose and lignin. These were dosed according to their content in the pretreated wheat straw resulting in the following concentrations: cellulose, 24 mg/mL; arabinoxylan, 2 mg/mL; lignin, 15 mg/mL; and pretreated wheat straw, 48 mg/mL. Both enzymes were added in a dosage of 500 nkat/mL - corresponding to 2.1 mg/mL protein for experiments with Cellic® CTec2 and 28.0 mg/mL protein for Novozym 188. The adsorption was evaluated by measurement of free β-glucosidase activity in the supernatant after incubation and comparison with a reference sample without substrate. The relative amount of free β-glucosidase activity after incubation with the different components can be seen in Figure 2. The experiment showed that for Novozym 188 there was no statistically significant adsorption of β-glucosidase activity to any of the tested compounds. By contrast, the β-glucosidases in Cellic® CTec2 adsorbed significantly to both lignin and pretreated wheat straw, which indicated that hydrophobic interactions between lignin and β-glucosidases in this enzyme preparation was most likely the cause of the adsorption onto the substrates. As the surface area of filter paper might be lower than that of powdered arabinoxylan or lignin, the adsorption to a powdered cellulose substrate (Avicel®) was also tested. The results revealed no statistical difference in adsorption of the two different β-glucosidases to the two substrates (data not shown).

We also tested commercial cellulase mixtures from other enzyme suppliers. These results confirmed that either the β-glucosidases did not adsorb to any of the tested components in pretreated biomasses or they adsorbed to pretreated wheat straw and lignin from pretreated wheat straw (unpublished data due to intellectual property rights). The adsorption of β-glucosidases to lignin was therefore not exclusively related to the β-glucosidase in Cellic® CTec2. Cellic® CTec2 and Novozym 188 seem to represent well extremes in the observed adsorption behavior for the β-glucosidases in a range of commercial cellulase preparations. Our results thereby emphasize that the β-glucosidases in today’s commercial cellulase preparations are not identical and therefore behave differently with regard to, for example, their adsorption behavior.

**Figure 3:**
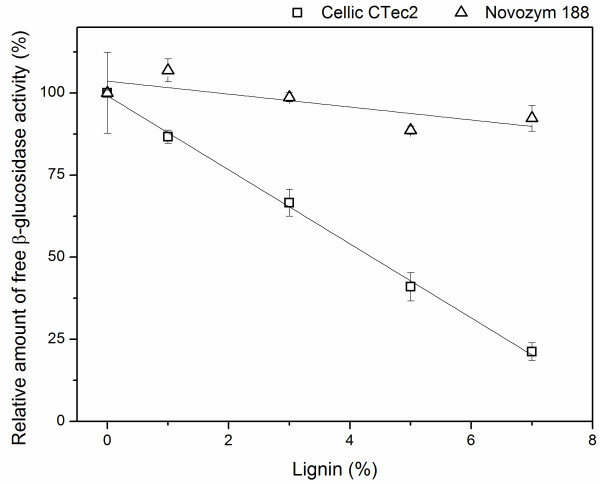
**Amount of free β-glucosidase activity in the presence of different amounts of enzymatically purified lignin from pretreated wheat straw.** Enzyme dosage 500 nkat/mL, lignin 0 to 7 w/w-% corresponding to approximately 0 to 100 mg/mL. The activities are relative to the activity in the control without added substrate for each enzyme.

The difference in lignin adsorption of β-glucosidases from Cellic® CTec2 and Novozym 188 was further emphasized when the concentration of lignin was varied. For Cellic® CTec2 there was a clear correlation (R^2^ = 0.98) between the amount of added lignin and the amount of free (and thereby also adsorbed) β-glucosidase activity (Figure 3). For Novozym 188 a small increase in adsorption was observed with increasing lignin concentration (the slope was different from null with a *P*-value of 0.0097). However, only the adsorption at 5% lignin was statistically different from the value without addition of lignin (Figure 3).

Here, it was interesting to note that a pretreated substrate containing 33% lignin (as the pretreated wheat straw used here) will correspond to around 7% lignin in a hydrolysis with initial 21% DM, which is a realistic concentration of DM in a commercial plant for production of advanced bioethanol. This means that the main part of the β-glucosidases will be adsorbed onto the pretreated biomass when using Cellic® CTec2 in production of bioethanol at high dry matter content, confirming the initial findings from experiments with hydrolysis and fermentation of pretreated wheat straw at 25% DM.

### Effect of biomass and pretreatment on β-glucosidase adsorption

Because lignin from different plant species differs in composition, for example, by the relative amount of monolignols, we tested if lignin from different pretreated biomasses adsorbed β-glucosidases differently. Furthermore, it is well-known the pretreatment also affects the lignin structure and therefore samples from different pretreatment methods were also included in the test.

**Figure 4:**
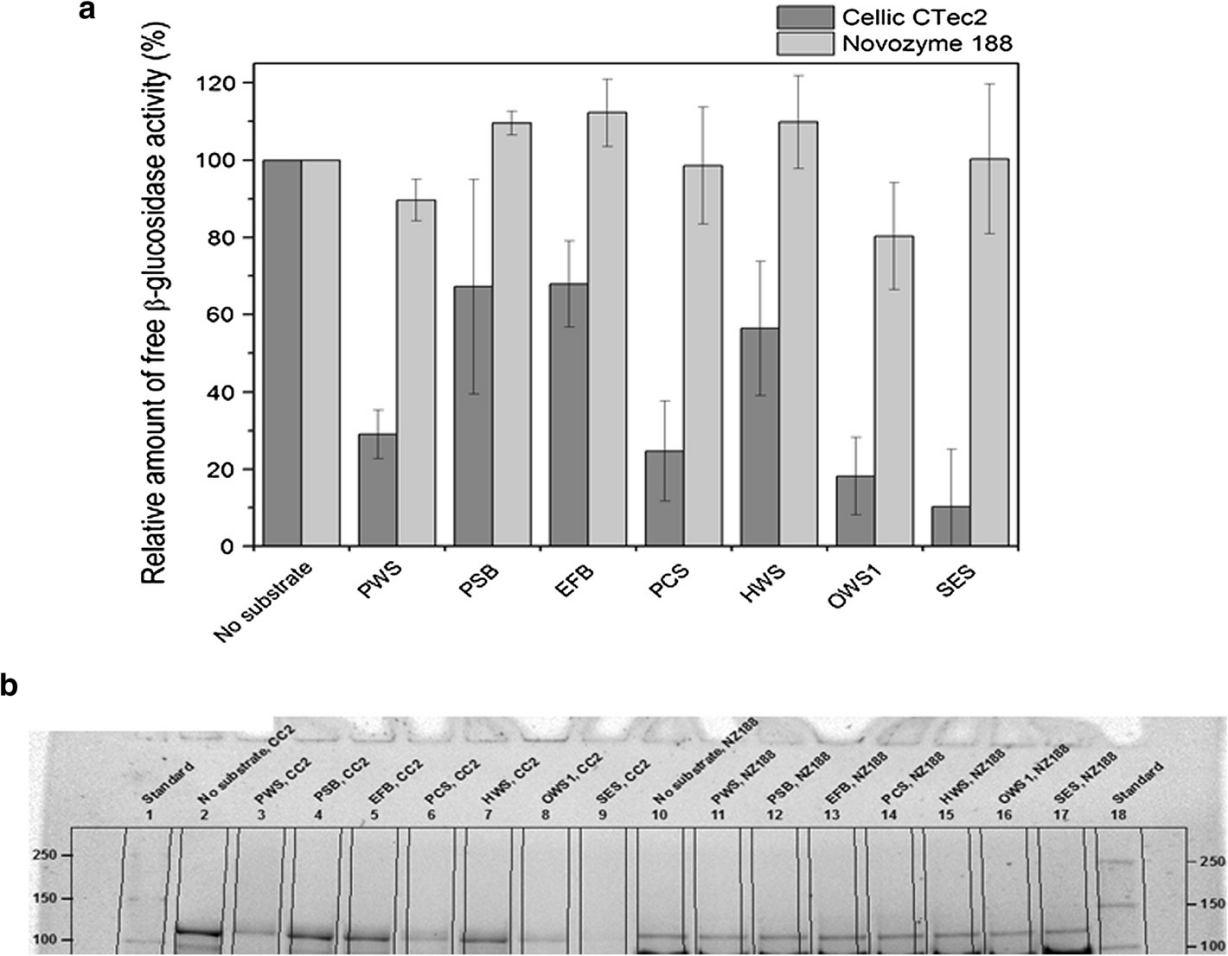
**Adsorption of β-glucosidase activity from Cellic® CTec2 (CC2) and Novozym 188 (NZ188) to different pretreated biomasses dosed to identical lignin content (1.5%). (a)** Relative amount of free β-glucosidase activity (relative to the activity in the controls for each enzyme). **(b)** Stain-free SDS-PAGE. The composition of the pretreated biomasses and their abbreviations can be seen in Table 1. Substrate loadings: No substrate (reference), 0 mg/mL; PWS (steam pretreated wheat straw), 48 mg/mL; PSB (steam pretreated sugarcane bagasse), 51 mg/mL; EFB (steam pretreated empty fruit bunches), 42 mg/mL; PCS (steam pretreated corn stover), 56 mg/mL; HWS (H_2_SO_4_ impregnated, steam pretreated wheat straw), 47 mg/mL; OWS1 (organosolv pretreated wheat straw), 129 mg/mL; and SES (steam exploded spruce), 31 mg/mL.

In the experiment, all biomasses were dosed according to their lignin content to obtain 1.5% lignin in the assay. The composition of the pretreated biomasses can be seen in Table 1. Comparison of the adsorption of β-glucosidases to different pretreated biomasses revealed that the adsorption of β-glucosidases from both of the tested enzyme preparations depended on both the pretreatment process and the type of biomass (Figure 4). Both the data for the relative amount of free β-glucosidase activity (Figure 4a) and the SDS-PAGE data (Figure 4b) confirmed that the adsorption of β-glucosidases from Novozym 188 to the different pretreated substrates was small or negligible. By contrast, β-glucosidases in Cellic® CTec2 adsorbed significantly but to a different extent to all the tested biomasses. Of all tested pretreated biomasses, steam-exploded spruce adsorbed the largest amount of the β-glucosidase activity. The reason for this may be the different composition of softwood lignin compared to that of the grasses. Softwood lignin contains mainly G-units, whereas grasses contain S, G and H units and are therefore structurally and chemically different [20].

**Table 1:** Composition of pretreated biomasses

					**Composition (% of dry matter)**						
**Abbreviation**	**Biomass**	**Origin**	**Pretreatment method**	**Pretreatment conditions (time (min)/temperature (°C))**	**Glucan**	**Xylan**	**Arabinan**	**Mannan**	**Galactan**	**Lignin**	**Ash**
PWS	Wheat straw	Denmark	Steam	14.8 / 195	51.0 ±0.4	4.1 ±0.2	0.0 ±0.0	-	-	33.0 ±0.3	5.1 ±0.1
PSB	Sugarcane bagasse	LA, USA	Steam	12.0 / 196	52.7 ±0.7	4.1 ±0.1	0.7 ±0.0	-	-	31.0 ±0.8	7.4 ±0.6
EFB	Empty fruit bunches	Malaysia	Steam	13.0 / 199	43.5 ±0.8	4.9 ±0.2	0.0 ±0.0	-	-	37.6 ±0.8	3.5 ±0.3
PCS	Corn stover	MN, USA	Steam	17.4/195	44.1 ±2.7	3.2 ±0.6	0.1 ±0.0	-	-	28.1 ±1.4	15.2 ±0.5
HWS	Wheat straw	Denmark	H_2_SO_4_ impregnation, steam	12.0/180	49.4 ± 0.9	4.5 ±0.2	1.0 ±0.2	-	-	33.3 ±0.6	5.4 ±0.2
SES	Spruce	Sweden	Steam explosion	5 to 7/210 to 215 + 2% to 3% SO_2_	38.5 ±0.3	2.3 ±0.2	0.4 ±0.0	3.5 ± 0.2	0.5 ± 0.1	50.6 ±1.5	1.0 ±0.3
OWS1	Wheat straw	Denmark	Organosolv	60/200 + 1% H_2_SO_4_	63.5 ±0.6	8.3 ±0.1	0.1 ±0.0	-	-	13.1 ±0.3	4.8 ±0.1
OWS2	Wheat straw	Denmark	Organosolv	60/200 + 1% H_2_SO_4_	60.7 ±1.1	7.2 ±0.0	0.1 ±0.0	-	-	20.6 ±0.4	3.0 ±0.1

The composition (in % of dry matter) of the pretreated biomasses is measured according to the National Renewable Energy Laboratory procedure for structural carbohydrates and lignin in biomass. [47].

Furthermore, our results demonstrated that the pretreatment method significantly affected the β-glucosidase adsorption. This was probably a result of the changes in especially the lignin structure that can occur during pretreatment, for example, from condensation [48,49], hydrolysis, formation of pseudolignin [50-53], sulfonation and solubilization of minor fragments, such as phenolic compounds.

The data also showed that for wheat straw, the pretreatment method significantly affected the adsorption of β-glucosidases from Cellic® CTec2 to the substrate. Organosolv pretreated wheat straw adsorbed significantly more activity than the two other types of pretreated wheat straw. Nakagame *et al*. have previously compared steam explosion and organosolv pretreatment of three different substrates, and their results also generally showed that organosolv pretreated substrates adsorbed more protein from commercial cellulases compared to steam exploded substrates [21]. Interestingly, even though Nakagame *et al*. [21] were working with a different enzyme system (a mixture of Spezyme CP and Novozym 188); their results supported our finding that organosolv pretreated substrates adsorbed more β-glucosidase activity and enzyme protein. Contrary to our findings, Pareek *et al*. [40] observed significant adsorption of β-glucosidase activity from Novozym 188 to different lignin preparations. They also noted that the descriptions of β-glucosidase adsorption in the literature are contradictory and the results seem to depend on the different structures of lignins, which is a result of, for example, different pretreatments and biomasses - as have also been described here.

**Figure 5:**
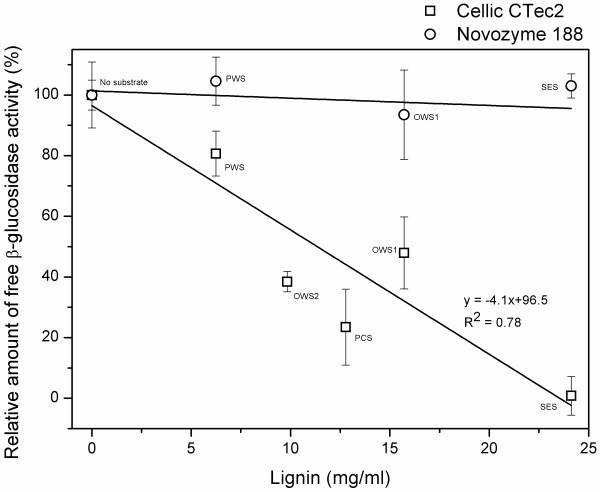
**Relative amount of free β-glucosidase activity from Cellic® CTec2 and Novozym 188 in the presence of different pretreated biomasses dosed to identical dry matter content.** The composition of the pretreated biomasses can be seen in Table 1. The pretreated biomasses were dosed according to their content of water insoluble solids (4.6% total DM). Lignin loadings: No substrate (reference), 0 mg/mL; PWS (steam pretreated wheat straw), 16 mg/mL; PCS (steam pretreated corn stover), 13 mg/mL; OWS1 and OWS2 (organosolv pretreated wheat straw at different pretreatment conditions), 6 mg/mL and 10 mg/mL; and SES (steam exploded spruce), 24 mg/mL. The activities are relative to the activity in the control without added substrate for each enzyme.

In the previous experiment with different pretreated biomasses, the amount of lignin (and thereby the ratio between lignin and β-glucosidase activity) was constant. This meant that because the lignin contents varied between the samples of pretreated biomass, the amount of DM added to each biomass also varied. Therefore, we also investigated if the DM content affected the β-glucosidase adsorption. The results of this experiment demonstrated that there was no correlation between the DM content and the β-glucosidase adsorption (data not shown). However, when the relative amount of free β-glucosidase activity was plotted as a function of the amount of lignin in the experiment with identical DM loading, there was a strong correlation between the lignin concentration in the experiment and the adsorption of β-glucosidases for Cellic® CTec2. Again, this was not the case for Novozym 188 (Figure 5). Furthermore, no clear correlation was found between β-glucosidase adsorption and the cellulose and hemicellulose concentration (results not shown).

The results also emphasized that lignin from different pretreatment methods and different biomasses do not behave identically as they did not adsorb identical amounts of β-glucosidase activity on a weight basis. However, by now it seems clear that some β-glucosidases adsorbed to lignin, and the adsorbed amount is determined by a combination of the pretreatment method and the type of biomass. Therefore, the choice of feedstock and process configuration will have a strong impact on the ability to recycle β-glucosidases through the liquid phase.

### Reduction of adsorption to lignin

The protein content and the β-glucosidase activity of the two tested enzyme preparations are very different. In other words, when dosing equal amounts of β-glucosidase activity (500 nkat/mL in the experiments) of Novozym 188 and Cellic® CTec2, much more protein is added in experiments with Novozym 188 compared to Cellic® CTec2 (2.06 mg/mL for Cellic® CTec2 and 28.0 mg/mL for Novozym 188).

To evaluate if the protein loading is the cause of the difference in adsorption behavior between β-glucosidases in the two preparations, an experiment was set up where the protein loading was identical (2.1 mg/mL), which mean that the β-glucosidase loading was approximately 500 nkat/mL for Cellic® CTec2 and 37 nkat/mL for Novozym 188. The results demonstrated that even at low protein loadings, the β-glucosidase in Novozym 188 did not adsorb to lignin (relative amount of free β-glucosidase activity without lignin: 100.0 ±3.0%; with lignin: 104.9 ±1.2%), contrary to the observations for Cellic® CTec2 (without lignin: 100.0 ±5.1%; with lignin: 65.7 ±3.7%).

**Figure 6:**
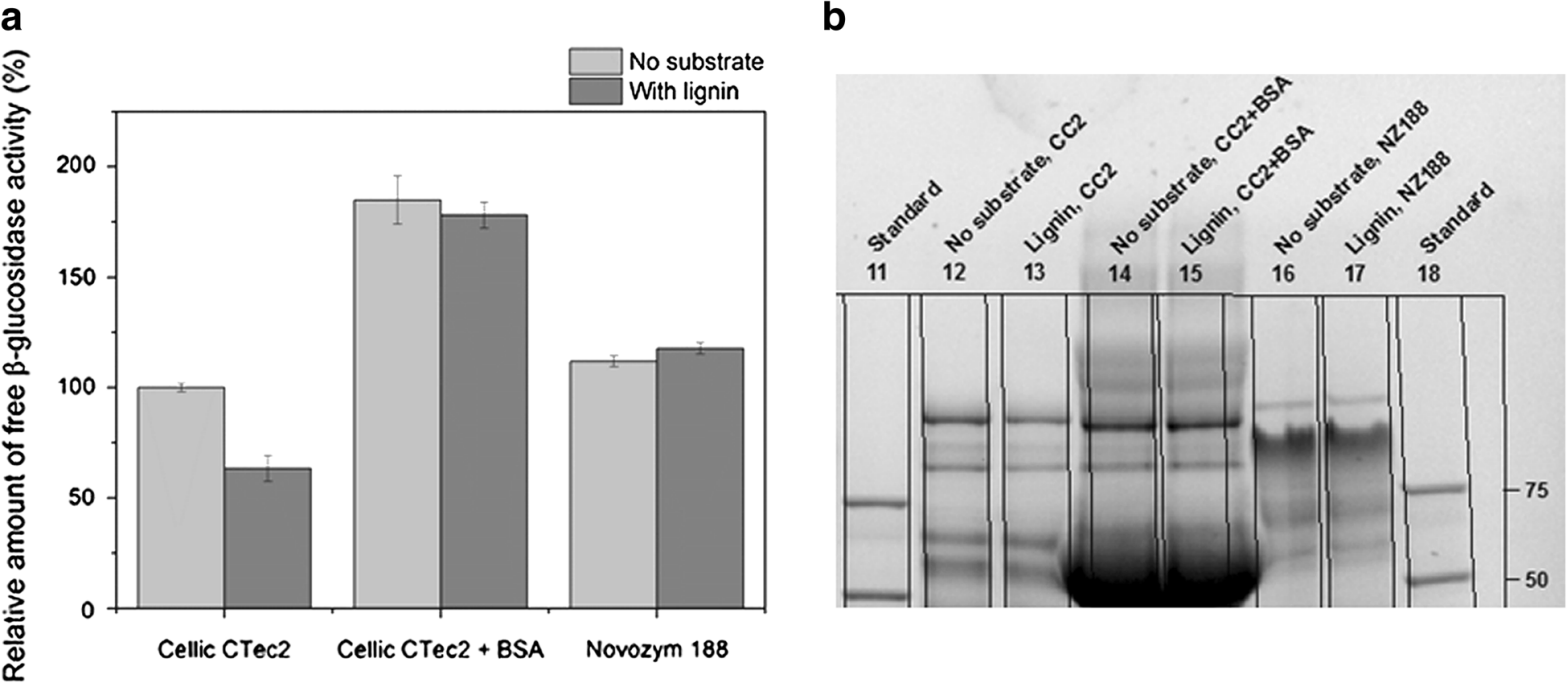
**The effect of protein concentration on adsorption of β-glucosidases to lignin for Cellic® CTec2 (CC2) and Novozym 188 (NZ188). (a)** Relative amount of free β-glucosidase activity and **(b)** stain-free SDS-PAGE. Total β-glucosidase activity 500 nkat/mL. Protein concentrations in experiment: Cellic CTec2, 2.1 mg/mL; Cellic CTec2 + BSA, 28.0 mg/mL; and Novozym 188, 28.0 mg/mL. The activities are relative to the controls without added substrate (and BSA) for each enzyme. BSA, bovine serum albumin.

Another experiment investigated the effect of the protein level on the β-glucosidase adsorption, comparing the activity of Cellic® CTec2 and Novozym 188 when Cellic® CTec2 was supplemented with protein in the form of bovine serum albumin (BSA) to obtain identical protein concentrations (28.0 mg/mL) as well as β-glucosidase activities (500 nkat/mL). This experiment showed that when supplementing Cellic® CTec2 with BSA to obtain a protein concentration equal to Novozym 188, addition of lignin did not affect the amount of free β-glucosidase activity (Figure 6a). This demonstrated that addition of BSA prevented nonproductive binding of β-glucosidase to lignin, possibly by preventing the hydrophobic interactions between β-glucosidases and lignin.

However, addition of BSA to solutions of enzyme also resulted in a large increase in the β-glucosidase activity for Cellic® CTec2 in the experiment without addition of lignin. This was also observed in SDS-PAGE where the intensity of the β-glucosidase band increased (Figure 6b). The reason for this might be that BSA prevents adsorption of β-glucosidases not only to lignin but also to other hydrophobic surfaces (in this case plastic from the centrifuge tubes) during the experiment, confirming that β-glucosidases from Cellic® CTec2 adsorbs significantly to hydrophobic surfaces. One could speculate if the adsorption phenomenon described in this paper is just due to adsorption to, for example, plastic and not biomass and lignin. However, all results presented here are relative to controls and should take unspecific adsorption to other surfaces than the added substrate into account. Furthermore, the clear correlation between adsorbed β-glucosidases and added lignin underlines that the observed phenomenon is indeed related to adsorption to lignin.

The experiments presented here showed that the adsorption of the β-glucosidase in Cellic® CTec2 could be prevented by adding BSA. Especially for cellulases, it has also previously been reported that addition of BSA can prevent unproductive or unspecific binding to lignin [33,54,55]. However, Yang and Wyman also report that addition of BSA reduced adsorption of β-glucosidases to lignin [33]. This prevention is believed to be through hydrophobic interactions between BSA and the pretreated biomass hindering the hydrophobic interactions between enzyme and lignin, which ultimately leads to an increased amount of free enzyme activity.

**Figure 7:**
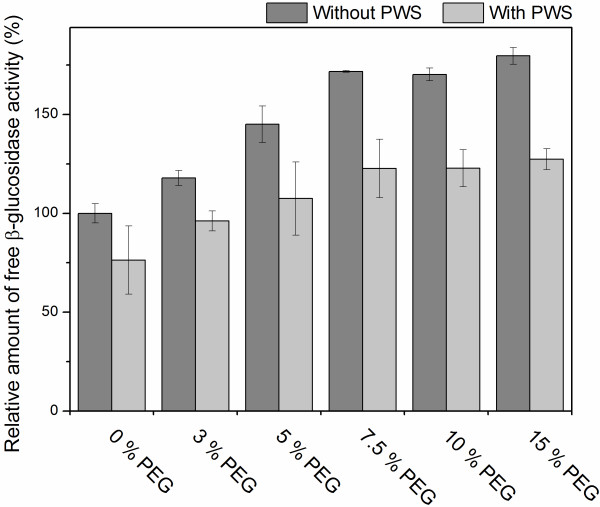
**Relative amount of free β-glucosidase activity from Cellic® CTec2 with or without addition of pretreated wheat straw in the presence of different concentrations of PEG6000.** Substrate loadings: without PWS, 0 mg/mL; with PWS, 48 mg/mL (1.5 w/v-% lignin, composition in Table 1). Total β-glucosidase activity: 500 nkat/mL. The activities are relative to the controls without added substrate. PWS, pretreated wheat straw.

Surfactants and surfactant precursors, such as Tween or poly(ethylene glycol) (PEG), have also been observed to increase the amount of free cellulase activity in the liquid phase [39]. Therefore, addition of protein or surfactants has been proposed to work by the same mechanisms when added to the hydrolysis of pretreated biomasses [54]. Most prior work with addition of for example, PEG has been focused on studying the effect on cellulases (endo- and exoglucanases) and not β-glucosidases. To confirm that addition of these types of compounds could also affect the adsorption of β-glucosidases from Cellic® CTec2. An experiment was carried out varying concentration of PEG6000 (0% to 15% of DM, PEG with an average molecular weight of 6000 g/mole) on pretreated wheat straw. The results showed that addition of PEG6000 could reduce the adsorption to lignin and increase the amount of free β-glucosidase activity by up to 45% (Figure 7). Furthermore, similar to the experiments with BSA, addition of PEG6000 to samples without substrate also increased the β-glucosidase activity. The results confirmed that PEG6000 can also prevent unproductive binding of the β-glucosidases in Cellic® CTec2 to lignin and possibly also other hydrophobic surfaces, which further supports the hypothesis that the β-glucosidase in Cellic® CTec2 interacts with lignin through hydrophobic interactions.

### Activity of adsorbed β-glucosidases

As described earlier, our experiments with hydrolysis and fermentation of pretreated wheat straw with Cellic CTec2 at 25% DM showed that although β-glucosidase activity was not found free in solution, cellobiose was not accumulating, indicating that the β-glucosidases were adsorbed but still catalytically active. This was confirmed by experiments where cellobiose was added to the solid residue after hydrolysis and fermentation of pretreated wheat straw. In these experiments, high concentrations of cellobiose (50 g/kg) were very rapidly hydrolyzed, confirming that the adsorbed β-glucosidases were indeed still catalytically active (data not shown).

To get a better understanding of the effect of the adsorption on the activity of the β-glucosidases, an experiment was set up comparing the activity of free and adsorbed β-glucosidases. This was done by comparing the activity of a β-glucosidase solution with and without addition of lignin and pretreated wheat straw. These data revealed that the adsorption had a small negative effect on the catalytic activity of the β-glucosidases from Cellic® CTec2 reducing this by approximately 15%. Addition of pretreated wheat straw reduced the activity to 85 ± 5% of original and lignin from pretreated wheat straw reduced the activity to 85 ± 8% (in both cases approximately 35% of the added activity was free in solution). This confirms the finding of Berlin *et al*. that the presence of lignin can have a small inhibitory effect (8% to 11% inhibition) on β-glucosidases caused by ionic-type or hydrophobic lignin enzyme interactions, even though they used a different β-glucosidase [56]. This inhibition/loss of activity does not seem to be a problem during hydrolysis and fermentation, since we rarely observe build-up of cellobiose even in experiments run at very high solids concentrations. The reason for this is likely that current commercial cellulase preparations contain large amounts of β-glucosidase activity to prevent product inhibition of the cellulase system - the batch of Cellic® CTec2 used here contained approximately 34,495 ± 2,935 nkat/g.

However, the β-glucosidase adsorption to lignin observed here was a problem when trying to recover and recycle the enzymes in Cellic® CTec2 through the liquid phase. In practice, a consequence of the adsorption is that recycling β-glucosidases by this method is not possible unless methods to efficiently desorb the β-glucosidases are identified. Otherwise, β-glucosidases can only be recycled if the residual solids are recycled. The implication of this is that not all β-glucosidases can be recycled since this would lead to build-up of lignin in the process.

## Conclusions

The results presented here show that there is distinct difference between how β-glucosidases can adsorb to lignin. The classical reference β-glucosidase from *A. niger* (from Novozym 188) does not adsorb significantly to lignin or biomass at the tested conditions, whereas the β-glucosidases in newer commercial cellulase preparations, such as Cellic® CTec2, adsorb significantly to lignin in pretreated biomasses. The adsorption to lignin depends on both pretreatment method and the type of biomass, that is, the nature and properties of the lignin. Adsorption could be reduced by addition of BSA or PEG6000 as adsorption to lignin or other surfaces is most likely due to hydrophobic interactions.

Adsorption of β-glucosidases to lignin is not *per se* a problem during hydrolysis and fermentation since the adsorption only seems to have a small negative effect on the catalytic activity. However, it represents a challenge when trying to recycle enzymes during the production of advanced bioethanol.

## Methods

### Raw materials

Wheat straw (Denmark), corn stover (MN, USA), empty fruit bunches from palm oil production (Malaysia), spruce (Sweden) and sugarcane bagasse (LA, USA) were used as raw materials for different pretreatment methods.

### Pretreatment methods

Three different pretreatment methods were used: steam pretreatment (with and without soaking in dilute H_2_SO_4_) in pilot scale at Inbicon A/S (Skærbæk, Denmark) [23]; steam explosion of SO_2_-impregnated biomass in pilot scale at SEKAB E-technology (Örnsköldsvik, Sweden) [57]; and organosolv pretreatment in laboratory scale at University of British Columbia (Vancouver, Canada) [58]. The composition of all the pretreated materials was measured using the National Renewable Energy Laboratory procedure for measuring structural carbohydrates and lignin in biomass [47]. The results of the analysis for the different pretreated biomasses and their pretreatment methods and conditions can be seen in Table 1.

Steam pretreatment of different biomasses (with and without H_2_SO_4_ impregnation) was done in the Inbicon pilot plant, which is capable of pretreating up to 100 kg/h biomass. The bales of biomass were mechanically cut into pieces of approximately 5 cm and fed to a mixer where biomass and soaking liquid (40 mg H_2_SO_4_ per kilogram of DM or water) were mixed to obtain approximately 35% to 40% DM. After mixing, the straw was fed into the pretreatment reactor. The residence time and temperature were varied to obtain pretreated material with the desired composition. After pretreatment, the material was washed (3 kg water per kilogram of DM in the biomass added to pretreatment).

Steam-exploded spruce was kindly provided by SEKAB E-technology. Spruce chips were impregnated with dilute sulfurous acid (SO_2_ in water, SO_2_: 2% to 3% of dry matter in feedstock) and pretreated at 210°C to 215°C for 5 to 7 min. After pretreatment, the slurry was centrifuged (10 min at 4200 *× g*) to collect the solids, which were used in the experiment described here.

For the organosolv pretreatment of wheat straw, the straw was first milled to pass a 10 mm screen. The pretreatment was done using a custom-built, four-vessel (2 L each) rotating digester made by Aurora Products Ltd. (Savona, BC, Canada) as previously described by Pan *et al*. [59] and Tu *et al*. [17]. In each vessel, 75 g dry weight straw and 525 mL cooking liquor (65% ethanol, 1% H_2_SO_4_) were mixed. The cooking time was 60 min at 200°C. The spent liquor was separated by vacuum filtration, and the pulp was washed three times with 300 mL of hot 65% ethanol (approximately 80°C). The washed pulp was homogenized in a standard British pulp disintegrator for 5 min. This material was then washed with 3 L of water.

### Enzymes

Novozym 188 and Cellic® CTec2 (both from Novozymes) were used for the adsorption experiments.

### Purification of lignin

Lignin from steam-pretreated wheat straw was purified enzymatically by hydrolyzing pretreated wheat straw from the Inbicon pilot plant (Skærbæk, Denmark) extensively with a mixture (5:1 v/v) of Celluclast 1.5 L and Novozym 188 (both from Novozymes); the enzyme loading was 75 filter paper units per gram of DM. After hydrolysis, the solid fraction was collected by centrifugation (3000 rpm for 10 min) and thoroughly washed. The solid material was re-suspended in phosphate buffer (pH 7.0) and treated with Alcalase® 2.5 L, type DX (Novozymes) to remove protein. After protein hydrolysis, the solid fraction (lignin) was collected and washed extensively before it was freeze-dried.

### β-glucosidase activity

The β-glucosidase activity was measured using *p*-nitrophenyl-β-d-glucopyranoside as substrate [60,61] using a method adjusted to 96-well microplates. First, the enzyme samples were diluted to concentrations within the standard curve with Na-citrate buffer (pH 4.8), that is, 0.056 to 0.556 nkat/mL. For samples containing particles, these were generally removed by centrifugation for 10 min at 4200 *× g* before dilution to avoid disturbance of the adsorption equilibrium. Twenty microliters of the enzyme solution and standards was transferred to a 96-well plate. Then 100 μl of 5 mM p-nitrophenyl-β-D-glucopyranoside (Sigma-Aldrich, St. Louis, MO, USA; cat. no. N7006) was added as substrate, and the plate was covered with self-adhesive foil and placed on a heating block at 50°C for 15 min. After incubation, 120 μl of stop solution (0.4 M glycine buffer, pH 10.8) was added along with 20 μl of diluted sample to the sample blanks before absorbance at 405 nm was read on a microplate reader (Multiscan™ FC, Thermo Fisher Scientific, Waltham, MA, USA). The measured β-glucosidase activity of Cellic® CTec2 was approximately 34,495 ± 2,935 nkat/g and for Novozym 188 was 3,117 ± 139 nkat/g.

### Protein measurement

Protein measurements were made using the ninhydrin assay with acidic hydrolysis of the proteins prior the ninhydrin assay, as previously reported [62].

All samples were analyzed with three repetitions. BSA (Sigma-Aldrich; cat. no. A7906) was used for the standard curve. The protein content of Cellic® CTec2 was 141 ± 6 mg/g and Novozym 188, 175 ± 6 mg/g.

### SDS-PAGE

SDS-PAGE was done in a Criterion system (BioRad, Hercules, CA, USA) using stain-free gels (Criterion TGX Stain-Free Precast Gels, 4% to 20%) with 18 wells and Tris/glycine/SDS buffer as running buffer. The gels were imaged and analyzed with a Gel Doc EZ system equipped with a stain-free tray. Samples containing Novozym 188 were diluted 1:19 (v/v), and samples with Cellic® CTec2 were diluted 1:1 (v/v) prior to sample preparation. This corresponded to protein concentrations of approximately 1.3 to 1.4 mg/mL if the enzymes had not adsorbed to the substrates. This was done by mixing 19 μl sample buffer (Laemmli sample buffer), 1 μl mercaptoethanol (reducing agent) and 20 μl of diluted sample. From this, 30 μl was loaded into each well on the gel. The gels were run at 200 V (constant) for approximately 45 min.

### Hydrolysis and fermentation of pretreated biomass at 25% DM

Pretreated wheat straw was hydrolyzed and fermented at a high DM content in a specially designed reactor employing the principle of free-fall mixing earlier described by Jørgensen *et al*. [63]. The DM content of pretreated wheat straw was adjusted to 25% (w/w) by addition of water before adjustment of pH to approximately 5.0 with Na_2_CO_3_. To some of the chambers, PEG 6000 (10 g per kilogram DM) was added before addition of different dosages of Cellic® CTec2. The biomass was hydrolyzed for 144 h at 40°C before cooling the fiber mash to 33°C and adding 1 g Thermosacc Dry per kilogram DM (Lallemand Biofuels & Distilled Spirits, Duluth, GA, USA ) and 4 g yeast extract (Merck, Darmstadt, Germany) for a total of approximately 200 h.

### Adsorption of β-glucosidases

The adsorption of β-glucosidase activity was estimated by subtracting the amount of free activity (measured in the supernatant by the β-glucosidase) from the amount of activity in reference samples without added substrate.

The experiments were conducted in 12 mL centrifuge tubes with 5 or 10 g total mass. All experiments were conducted in 50 mM Na-citrate buffer at pH 4.8 to avoid changing the surface charge of lignin. During the experiment buffer, substrate and enzyme were dosed and thoroughly mixed, and the tubes were incubated at 33°C at 250 rpm for 1 h. After incubation, all tubes were centrifuged at 4200 *× g* for 10 min, and the supernatant was removed and used for analysis of free β-glucosidase activity. The supernatant was diluted with Na-citrate buffer at pH 4.8 prior to measurements; typically, the dilution factor for samples from experiments with Cellic® CTec2 was 800 to 1,200 and for samples with Novozym 188 was 1,000 to 1,500. Unless otherwise stated, both enzymes were in all experiments dosed to a concentration of 500 nkat/mL of liquid based on the activity of the preparation. This corresponds to approximately 2.1 mg protein per milliliter for Cellic® CTec2 and 28.0 mg/mL for Novozym 188. A description of the details of the individual adsorption experiments can be seen in the following paragraphs.

### β-glucosidase adsorption to pretreated wheat straw

Different substrates were used to mimic the constituents of pretreated wheat straw; these were cellulose (filter paper or Avicel®, PH-101, Sigma Aldrich; cat. no. 11365), hemicellulose (arabinoxylan, Megazyme, Bray, Co. Wicklow, Ireland, cat. no. P-WAXYI), purified lignin from pretreated wheat straw (see earlier description of purification procedure) and pretreated wheat straw (for compositional data see Table 1). These substrates were dosed according to their content in the pretreated wheat straw when dosing this to a total lignin loading of 1.5% (w/w). The concentrations of the substrates were: cellulose, 24 mg/mL; hemicellulose, 2 mg/mL; lignin, 15 mg/mL; and pretreated wheat straw, 48 mg/mL.

The effect of different concentrations of enzymatically purified lignin on the amount of free β-glucosidase activity was also tested. The experiment was carried out with lignin loadings of 0% to 7% lignin (w/w) - corresponding to lignin concentrations in the range of 0 to 100 mg/mL.

### Effect of biomass and pretreatment on β-glucosidase adsorption

To test if lignin from different pretreated biomasses adsorbed β-glucosidase activity differently, the different biomasses were all dosed according to their lignin content to a loading of 1.5% lignin (w/w); the composition of the pretreated biomasses and abbreviations can be seen in Table 1. The added amounts were: PWS, 48 mg/mL; PSB, 51 mg/mL; EFB, 42 mg/mL; PCS, 56 mg/mL; HWS, 47 mg/mL; OWS1, 129 mg/mL; and SES, 31 mg/mL.

We also tested the effect of adding identical amounts of water-insoluble solids from the different pretreated biomasses. Here, 4.55 w/w-% DM was added to the experiment, resulting in different lignin concentrations: PWS, 16 mg/mL; PCS, 13 mg/mL; OWS1, 6 mg/mL; OWS2, 10 mg/mL; and SES, 24 mg/mL.

### Reduction of adsorption to lignin

The effect of protein loading, adding identical amounts of protein (2.1 g/mL) of the two enzyme preparations, was also tested. This resulted in different β-glucosidase loadings: Cellic® CTec2, 500 nkat/mL; Novozym 188, 37 nkat/mL. The experiment was carried out with 3 w/w-% purified lignin (96 mg/mL).

The effect of the protein addition on the β-glucosidase adsorption to lignin was investigated by dosing identical amounts of β-glucosidase activity (500 nkat/mL) of the two enzymes and then supplementing Cellic® CTec2 with BSA (Sigma-Aldrich; cat. no. 7906) to reach identical protein loadings (28 mg/mL). The experiment was carried out at 3 w/w-% lignin from either purified lignin or pretreated wheat straw giving a substrate loading of 32 mg/mL for lignin and 96 mg/mL for pretreated wheat straw.

To investigate the potential for reducing the adsorption of β-glucosidase from Cellic® CTec2 to lignin, we investigated the effect of adding different concentrations of PEG6000 with an average molecular weight of 6000 g/mole to pretreated wheat straw. The experiment was carried out with steam-pretreated wheat straw at 1.5 w/w-% lignin (48 mg/g) and with concentrations of PEG6000 between 0% and 15% of lignin (0 to 7.2 mg/g).

### Activity of adsorbed β-glucosidases

The effect of adsorption to lignin of the β-glucosidases was evaluated by measuring the β-glucosidase activity of the suspension of lignin and Cellic® CTec2 after incubation without prior separation into liquid and solid phase. Since the samples were heavily diluted (dilution factors between 800 and 1,200) before measurement of β-glucosidase activity, the presence of particles did not affect the assay. The experiment was carried out at 1.5 w/w-% lignin from either purified lignin or pretreated wheat straw.

### Statistical analyses

All statistical analyses were performed using GraphPad Prism version 5.04 for Windows (GraphPad Software, San Diego, California, USA).

## Abbreviations

BSA: Bovine serum albumin; CC2: Cellic® CTec2; DM: Dry matter; EFB: Steam-pretreated empty fruit bunches; HWS: H_2_SO_4_-impregnated steam-pretreated wheat straw; NZ188: Novozym 188; OWS: organosolv-pretreated wheat straw; PCS: Steam-pretreated corn stover; PEG: Poly(ethylene glycol); PEG6000: Poly(ethylene glycol) with an average molecular weight of 6000 g/mol; PSB: Steam-pretreated sugarcane bagasse; PWS: Steam-pretreated wheat straw; SES: Steam-exploded spruce.

## Competing interests

MØH is employed as an industrial PhD student at DONG Energy, who may have a financial interest in this work. HJ declares no competing interests.

## Authors’ contributions

MØH and HJ conceived and designed various aspects of the experiments. MØH carried out the experiments, analyzed the results and drafted the manuscript; HJ helped write and review the manuscript. Both authors read and approved the final manuscript.
